# Social sciences: vital to improving our understanding of health equity, policy and systems

**DOI:** 10.1186/s12939-017-0546-6

**Published:** 2017-04-03

**Authors:** Karen Daniels, Johanna Hanefeld, Bruno Marchal

**Affiliations:** 1 Health Systems Research Unit, Cape Town, South Africa; 2 Health Policy and Systems Division, School of Public Health and Family Medicine, University of Cape Town, Cape Town, South Africa; 3 Health Services Organisation unit, Department of Public Health, Institute of Tropical Medicine, Antwerp, Belgium

**Keywords:** Social science, Health policy and systems research, Equity, Methods

Founded by the International Society for Equity in Health 15 years ago, the International Journal for Equity in Health (IJEqH) has from its inception been committed to redressing not just health inequity, but also inequity in the publishing of research on health cb [1]. An editorial marking the society’s 10^th^ anniversary remarked that “In particular the conferences and the journal have both provided a voice to researchers from low and middle income countries, giving life and embodiment to the values of inclusion, action-based research, research-to policy processes, and the vital role of civil society in strengthening action for health equity.” [1]. This commitment to giving voice to diversity was clear to the members of SHAPES (Social science approaches for research and engagement in health policy & systems) a thematic working group of Health Systems Global [2], when, in July 2016, the Journal published an open letter from our members and affiliate group members, critiquing the lack of publishing space given to qualitative research in many academic journals [3]. Our critique focused on how methodological gatekeeping within mainstream publishing privileged certain voices in global health over others, and in so doing, was in effect silencing many researchers in low- and middle- income countries (LMICs) [3]. Such gatekeeping limits the range of publicly accessible knowledge on solutions to health problems experienced in LMIC settings [3] and these inequitable publishing practices contribute to inequitable knowledge distribution about solutions to health problems faced by the most marginalised populations on the globe.

Social science approaches are firmly embedded in the field of health policy and systems research (HSPR), where they not only shape our methods of enquiry, but provide key critical theory through which we are better able to understand the people and the processes shaping health systems [4]. The need for a greater emphasis on social science theory and methods as a means to answer some of the core questions in the emerging field of HPSR has been established during the past decade [5]. Finding solutions to contemporary challenges in health systems, to achieving Universal Health Coverage (UHC) and the Sustainable Development Goals (SDGs), will indeed not occur through application of traditional quantitative research methods and techniques alone, as these methods do not take into account the perspectives of the people at the forefront of implementation.

Research on the SDGs will need to be far more sophisticated than assessing target indicators through numerical measures; instead, this research will have to embrace complexity, and social science approaches are well suited to the task [6]. Because qualitative approaches take into account the experience of policy actors, they may serve to tell the real life stories of the SDG implementation process. Through these stories, we may be better able to understand the mechanisms through which equity is enhanced or inequity is further entrenched [7].

Research methods born out of the positivist paradigm artificially separate the research subject and object [5], which limits our capacity for observations on the dynamic and the interactive processes that occur between patients, providers, managers, policymakers, communities and activists. These interactions are the heart of health systems [5]. Moreover, notions of research objectivity limit the field, especially when it comes to innovative research practice. Participatory research methods, which have contributed significantly to insights in the field of health systems, have demonstrated the value of action research and active engagement by researchers in the systems they seek to study [8]. Health policy and systems researchers cannot simply observe the health systems at a distance; for change to occur, we need to engage with the actors in the system, and in so doing we become actors ourselves [9].

The outbreak of Ebola Virus Disease (EVD) in West Africa from 2014 to2016 highlighted the importance of health systems and their resilience to disease outbreaks and other health emergencies [10]. The recent symposium on Health Systems Research (HSR), held in Vancouver in 2016 reflected this focus with a theme on responsive and resilient health systems [11]. While the global health community is still reflecting on the lessons from the Ebola outbreak, a lesson shared by researchers, activists and implementers at the HSR Symposium was the key role of the connection between communities, including community health workers, and the more formal health system, in responding to Ebola. Critically, the role of social science, including that of anthropologists and sociologists in enabling such a connection, was recognised as key in this. The outbreak of Ebola Virus Disease in West Africa underscored the importance of social science in general, and of qualitative research in particular when it comes to practices and research focused on health policy and systems. This suggests that we can only deepen our understanding and rise to challenges in health policy and systems through an increased emphasis on rigorous qualitative social science research within the field of HPSR. Achieving policy goals such as UHC and the wider SDG three on greater health and well-being, will be impossible without this shift in focus.

SHAPES is dedicated to promoting and supporting innovative research methodology suitable to the realities of health policy and systems research. Unfortunately, our experiences have shown there are many challenges to publishing research conducted using social science approaches as qualitative research is not always well received by mainstream journals. In the open letter published in this journal in 2016, we detailed the contribution of qualitative research to health policies and health systems globally [3]. Continuing in this spirit, SHAPES commits to collaborate with the IJEqH in establishing a thematic series that will both profile social science approaches to health policy and systems research, as well as seek to build capacity through mentoring. It is envisioned that capacity building and mentorship will happen at three levels – authorship, peer reviewing, and the training of first time academic editors. The thematic series will also showcase good examples of social science approaches to health policy and systems questions, and present particularly innovative methods that may not easily get recognition in mainstream journals. The IJEqH’s commitment to equity is not only in subject matter, but also to equity in the range of articles, authors, reviewers and guest editors so that researchers, policy makers and practitioners from high, middle and low income countries can participate.. We therefore see this as an opportunity to encourage and support submissions from authors from all over the globe, and to work together to ensure the publication ofh high quality papers.

As SHAPES, we very much look forward to this collaboration and once again congratulate the editorial team and everyone who has contributed to the success of the journal over the last 15 years. We are hopeful that our collaboration around a thematic series will lead to even great success over the next 15 years.
